# From Ethnomedicine to Plant Biotechnology and Machine Learning: The Valorization of the Medicinal Plant *Bryophyllum* sp.

**DOI:** 10.3390/ph13120444

**Published:** 2020-12-04

**Authors:** Pascual García-Pérez, Eva Lozano-Milo, Mariana Landin, Pedro P. Gallego

**Affiliations:** 1 Applied Plant & Soil Biology, Plant Biology and Soil Science Department, Biology Faculty, University of Vigo, E-36310 Vigo, Spain; pasgarcia@uvigo.es (P.G.-P.); elozano@alumnos.uvigo.es (E.L.-M.); 2 CITACA—Agri-Food Research and Transfer Cluster, University of Vigo, E-32004 Ourense, Spain; 3 Pharmacology, Pharmacy and Pharmaceutical Technology Department, Grupo I+D Farma (GI-1645), Pharmacy Faculty, University of Santiago, E-15782 Santiago de Compostela, Spain; m.landin@usc.es; 4 Health Research Institute of Santiago de Compostela (IDIS), E-15782 Santiago de Compostela, Spain

**Keywords:** *Bryophyllum*, traditional medicine, secondary metabolism, bioactive and phenolic compounds, bufadienolides, antioxidants, cytotoxic activity, plant tissue culture, artificial intelligence

## Abstract

The subgenus *Bryophyllum* includes about 25 plant species native to Madagascar, and is widely used in traditional medicine worldwide. Different formulations from *Bryophyllum* have been employed for the treatment of several ailments, including infections, gynecological disorders, and chronic diseases, such as diabetes, neurological and neoplastic diseases. Two major families of secondary metabolites have been reported as responsible for these bioactivities: phenolic compounds and bufadienolides. These compounds are found in limited amounts in plants because they are biosynthesized in response to different biotic and abiotic stresses. Therefore, novel approaches should be undertaken with the aim of achieving the phytochemical valorization of *Bryophyllum* sp., allowing a sustainable production that prevents from a massive exploitation of wild plant resources. This review focuses on the study of phytoconstituents reported on *Bryophyllum* sp.; the application of plant tissue culture methodology as a reliable tool for the valorization of bioactive compounds; and the application of machine learning technology to model and optimize the full phytochemical potential of *Bryophyllum* sp. As a result, *Bryophyllum* species can be considered as a promising source of plant bioactive compounds, with enormous antioxidant and anticancer potential, which could be used for their large-scale biotechnological exploitation in cosmetic, food, and pharmaceutical industries.

## 1. Introduction

The genus *Kalanchoe* (Adanson, 1736 [1]) belongs to the Crassulaceae family and comprises 150 to 200 succulent species native to Madagascar and naturalized across Africa, South America, and Asia [2,3]. Kalanchoe constitutes a complex genus with an intricate taxonomy, not yet clearly elucidated. Two different trends have been remarkable throughout the published literature concerning both its nomenclature and systematics [4]. Authors disagree whether the classification is based on a single genus called *Kalanchoe* (sensu lato) or three separate sections: *Kalanchoe* (sensu stricto), *Bryophyllum* Kahl. (Salisbury, 1805 [5]), and *Kitchingia* (Baker, 1881 [6]). However, other authors propose a three-subgenera classification of the genus *Kalanchoe*, due to different evolutive arguments, morphological traits [7] and molecular analyses [8], including *Kalanchoe*, *Bryophyllum* and *Calophygia* [4]. Amongst the different subgenera, the subgenus *Bryophyllum* includes around 25 species, endemic to Madagascar [9] that gained much interest on plant science research, as they are considered model plants for different physiological features: the Crassulacean Acid Metabolism (CAM) [10], vegetative reproduction [11], plant cell regeneration [12], and a source of therapeutical compounds [13]. Nevertheless, the most relevant feature associated to this subgenus is the use of their constitutive species in the traditional medicine worldwide, thus considering *Bryophyllum* sp. as medicinal plants, due to their associated bioactivities [13].

**Figure 1:**
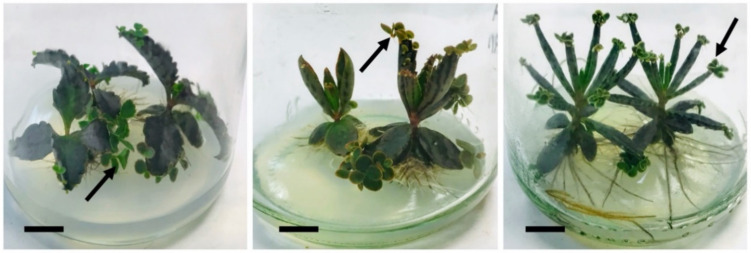
In vitro-cultured plants of *B. daigremontianum* (**left**); *B. × houghtonii* (**center**); and *B. tubiflorum* (**right**). Bars = 1 cm; arrows indicate plantlets formed asexually on leaf margins. Original figure.

CAM photosynthesis is an advantageous adaptative strategy that enables plant adaptation to arid ecosystems, as it is the case of the whole Kalanchoe genus [14]. *Bryophyllum* species present a flexible CAM regime, with no time restriction on CO_2_ uptake, which is fixed at night [15]. On the other hand, *Bryophyllum* sp. present a highly specialized asexual reproductive mechanism, based on the symmetric plantlet development along the leaf margins or leaf tips of adult plants (Figure 1) [12,16]. Such clonal-spreading reproductive mechanism is driven by a complex phenomenon that combines both embryogenic and organogenetic events that has not been fully elucidated to date [17,18,19,20,21]. Both the metabolic and reproductive patterns found on *Bryophyllum* sp. contribute to the invasiveness of these species. It allows them a rapid colonization of unexplored territories with high adaptative efficiency, which has contributed to their worldwide naturalization [22,23].

*Bryophyllum* and other Kalanchoe species have been widely used in the traditional medicine of vast regions throughout Africa, South America, and Asia [24]. Because of its wide distribution and ubiquitous medicinal use, much research on this subgenus has focused on *Bryophyllum pinnatum* (Lam.) Oken [25,26,27]; however, there is an extensive variety of other species that have also been exploited in Ethnomedicine, such as: *B. daigremontianum* (Raym.-Hamet et Perr.) Berg. [28], *B. tubiflorum* Harv. [29,30], and *B. × houghtonii* D.B. Ward (syn. *B. daigremontianum × tubiflorum*) [31]. Leaf and root-derived formulations have been mostly used for the treatment of several common illnesses such as burns, wounds, insect bites, skin diseases, cough, fever or several infections, and chronic diseases, such as diabetes, and neurological and neoplastic diseases (Table 1).

The great therapeutic potential reported on *Bryophyllum* sp. [39] has promoted in-depth phytochemical analysis to adequately evaluate its biological and pharmacological properties [55,56]. Several authors have demonstrated the whole bioactive potential of *Bryophyllum*-derived extracts, acting as multifaceted agents.

The anti-inflammatory activity of *Bryophyllum* extracts has been determined by different methods using both in vivo and in vitro models. For instance, aqueous extracts from *B. pinnatum* were shown to exert a relevant effect against croton oil-induced ear edema and carrageenan-induced paw edema in murine models, driven by a decrease in pro-inflammatory cytokines [57]. Moreover, different flavonoids produced by *B. tubiflorum* showed an inhibitory effect on nitric oxide production by lipopolysaccharide-induced macrophage in vitro RAW264.7 cell line [58].

The antimicrobial activity attributed to *Bryophyllum* extracts was shown to present a high effectiveness against a wide range of both bacterial and fungal activities. In this sense, hydroethanolic extracts from *B. fedtschenkoi* showed a strong inhibitory effect against different antimicrobial resistant strains from the ESKAPE complex, including both Gram-negative and Gram-positive bacteria [37]. Similarly, the bactericidal effect of *B. crenatum* leaf juice against *Bacillus subtilis* and *Klebsiella pneumoniae* was also reported, as well as high effectiveness of methanol extracts from *B. pinnatum* to Gram-positive bacteria [34]. Moreover, different isolated fractions from *B. daigremontianum* ethanolic extracts promoted a potent activity against Safase S-04 yeast strain, fungi, such as *Candida albicans* and *Aspergillus niger*, and bacteria, including *Staphylococcus aureus* and *Escherichia coli* [59]. Furthermore, the antiviral activity of *Bryophyllum* extracts has been also assessed for relevant viral diseases. It is the case of the antiviral activity of kaempferol derivatives from *B. daigremontianum* against Herpes Simplex Virus (HSV) types 1 and 2 [60] and bryophyllin B from *B. pinnatum* as a potent inhibitor of Human Immunodeficiency Virus (HIV) [61].

Additionally, the analgesic and sedative properties of *Bryophyllum* extracts were evaluated using in vivo murine models, indicating that leaf extracts from *B. crenatum* showed a protective effect against formalin and acetic acid-induced pain and inhibited the manifestation of seizures under convulsant agents application [32].

The antioxidant properties of *Bryophyllum* extracts have been widely reported by a plethora of different methods. The radical scavenging activity against 2,2-diphenyl-picryl-hydrazyl (DPPH), superoxide anion and nitric oxide of *B. daigremontianum*, *B. tubiflorum*, *B. × houghtonii,* and *B. pinnatum* leaf and aerial part extracts was reported [62,63]. The inhibition of lipid peroxidation by hydromethanolic extracts from aerial parts of *B. daigremontianum*, *B. tubiflorum*, and *B. × houghtonii*, cultured in vitro was also determined [64]. Moreover, cell-based in vitro antioxidant assays have been performed for the inhibition of lipid peroxidation of root extracts from *B. daigremontianum* [24].

*Bryophyllum* extracts have been also shown to present insecticidal properties, as a consequence of bufadienolide production, as reviewed later. In this sense, methanolic leaf extracts from *B. daigremontianum*, *B. pinnatum,* and *B. × houghtonii* showed an intense effect against silkworm larvae (*Bombyx mori*) [65,66,67].

Moreover, cardioprotective and antihypertensive properties were attributed to different *Bryophyllum* sp. [68]. For instance, the aqueous extracts of *B. pinnatum* have been shown to exhibit in vivo antihypertensive activity on high salt-loaded rats models [69]. Furthermore, isolates from *B. daigremontianum* root extracts developed an in vitro anti-thrombotic activity [70].

Against all the bioactivities associated with *Bryophyllum* sp., the cytotoxic activity gained much interest during the phytochemical characterization of these species [71]. A great variety of in vitro models have been employed for the determination of cytotoxic and anti-cancer activities on different *Bryophyllum* species, whose extracts have been tested against a high number of cancer cell lines [13,68]. Due to the relevance of this bioactivity, the cytotoxic properties of *Bryophyllum* extracts are included during this review.

Finally, there are additional health-enhancing properties related to *Bryophyllum* sp., as it is the case of hepatoprotective, antidiabetic activities. Thus, the leaf juice and aqueous of *B. pinnatum* showed a marked in vivo hepatoprotective effect on carbon tetrachloride-induced hepatotoxicity in rats [72], as well as hypoglycemic and hypocholesterolemic effects in streptozotocin-induced diabetic rats [73].

As a result, the combination of all bioactivities attributed to *Bryophyllum* sp. aroused the interest in the study of their great therapeutic potential, which is a challenge, as it is an unexplored subgenus with countless potential as a health promoter. This is a systematic review in which general search engines, including PubMed, the Web of Science, and Google Scholar were employed, according to Preferred Reported Items for Systematic Reviews and Meta-Analyses (PRISMA) guidelines.

## 2. *Bryophyllum* sp. Secondary Metabolites as Antioxidants and Anticancer Agents

It is now well-known that the full set of bioactivities attributed to *Bryophyllum* sp. is developed by a plethora of phytoconstituents, including phenolic compounds, bufadienolides, organic salts, terpenoids and fatty acids [55]. Phytoconstituents are considered secondary metabolites, since they are biosynthesized by induction of secondary metabolism, which is responsible for the defensive and adaptative plant response against environmental threads and biotic stress [74,75]. Phenolic compounds and bufadienolides are considered the two main families of secondary metabolites of *Bryophyllum* sp., widely distributed throughout the subgenus [13]. Furthermore, they are responsible for the bioactivity associated with *Bryophyllum* sp. and, consequently, a deeper insight into these compounds will be provided.

### 2.1. Phenolic Compounds

Two major subfamilies of phenolic compounds have been widely reported for *Bryophyllum* sp.: phenolic acids and flavonoids [76,77], which have been recently found to accumulate inside highly specialized leaf cells, called idioblasts [78].

The antioxidant activity of *Bryophyllum* phenolic compounds, focused on the free-radical scavenging activity, has been largely determined [63,79]. Recently, the antioxidant capacity of *Bryophyllum* extracts for preventing the lipid oxidation of omega-3 enriched fish oil emulsions was reported, thus conferring a valuable approach for the application of *Bryophyllum*-derived by-products in the food and pharmacological industries [64]. In the same way, the polyphenols from *Bryophyllum*-derived extracts may be efficiently purified using environmental-friendly procedures, like the use of activated carbon [80]. These approaches have been developed in order to allow the industrial exploitation of *Bryophyllum* polyphenols, due to the increasing interest in the research of these medicinal plants.

The great diversity of bioactivities described for these compounds places the phenolic compounds of *Bryophyllum* sp. as one of the main families of plant secondary metabolites that boost the phytochemical potential of this subgenus [62,64,81].

#### 2.1.1. Phenolic Acids

Three species of *Bryophyllum* present high content in phenolic acids: *B. pinnatum*, *B. daigremontianum,* and *B. tubiflorum*, mostly located in leaf tissues (Table 2) [76,82]. Both subfamilies of phenolic acids have identified compounds in either free or glycosylated forms. Caffeic acid and ferulic acid are the most abundant cinnamic acids, while within the benzoic acids it is protocatechuic acid. β-resorcylic and γ-rosorcylic acids have also been referenced, although these are more unusual. [63].

Concerning bioactivities, phenolic acids are considered powerful antioxidants whose activity depends on the number, position, and combination of hydroxyl groups within their structure [83]. Potential therapeutic properties for them have also been reported, as they promote antimicrobial, antiviral, cytotoxic, and anti-inflammatory activities [84,85,86,87]. Phenolic acids from *Bryophyllum*-derived extracts have already been related to the development of antibacterial and antifungal activity against a series of pathogenic microorganisms [88], antioxidant activity, and cytotoxicity against human lymphoblastic leukemia J45 and H9 T-cell lines [63].

#### 2.1.2. Flavonoids

Flavonoids are universally found in *Bryophyllum* sp. in *O*-glycosylated form. To a large extent, they have been reported in three species, namely: *B. pinnatum*, *B. daigremontianum* and *B. tubiflorum* (Table 3). The flavonol glycosides were shown as the most abundant subfamily of flavonoids, showing a restricted accumulation on leaf tissues [13,76,90]. Both kaempferol and quercetin glycosides were found in *Bryophyllum* species [39,94,95]. Other flavonoid subfamilies, such as flavones and catechins, have also been reported, and a number of anthocyanins have been isolated from the flowers of different species [39,96], which are stored in the foliar idioblasts of *B. daigremontianum* [82] and *B. tubiflorum* [78].

The antioxidant activity of flavonoids is directly proportional to the number and position of hydroxyl groups in their structure [97], that assist in the dissipation of electrons generated after UV-overexposure [98]. Additionally, they also prevent lipid peroxidation [99] (by decomposing lipid peroxides and scavenging harmful free-radicals) and develop an effective metal chelation activity [100]. The free-radical scavenging [62,101,102] and lipid oxidation preventing activities [64] of *Bryophyllum*-derived extracts rich in flavonoids have already been reported. Other bioactivities, such as antibacterial [103], antiviral [104], cytotoxic [105], anti-inflammatory [106], cardioprotective [107], sedative and anti-diabetic activities [108] have been associated to flavonoids. These bioactivities have been extensively studied for *Bryophyllum* sp. and have also been related to flavonoid content, mainly using *B. pinnatum* as a plant model [88,89,94,95,109,110].

### 2.2. Bufadienolides

**Figure 2:**
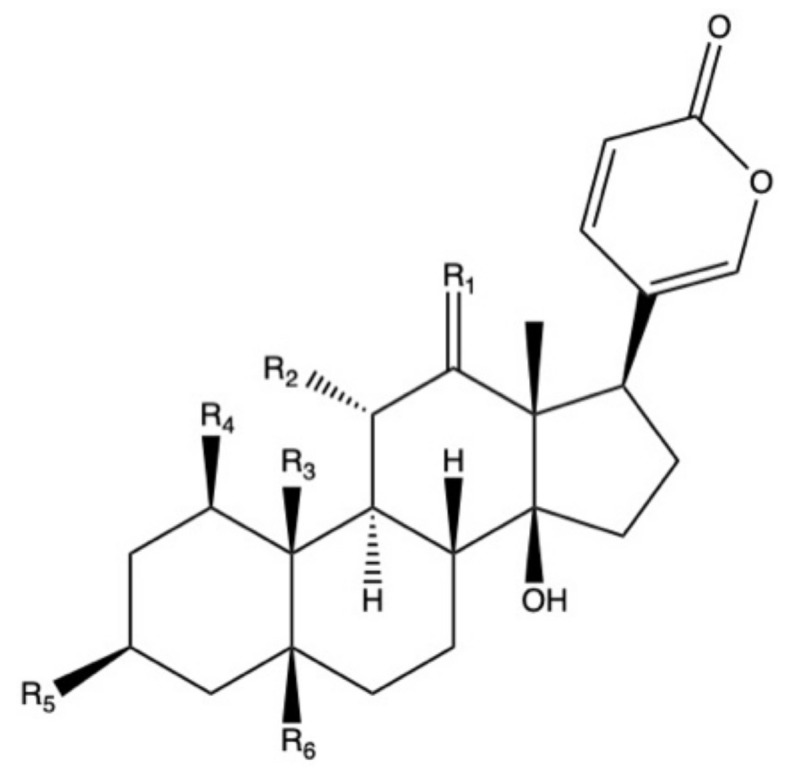
Basic molecular structure of bufadienolides.

Bufadienolides constitute a subfamily within cardiac glycosides family of secondary metabolites and they are considered polyhydroxy C-24 steroids, presenting an α-pyrone ring at the C-17β position (Figure 2) [113]. Bufadienolides presence in *Bryophyllum* species is genotype and organ dependent [68], being four species the most representative sources of these compounds: *B. daigremontianum*, *B. × houghtonii*, *B. tubiflorum,* and *B. pinnatum* (Table 4). Universally-distributed bufadienolides, such as bersaldegenin and bryophyllin derivatives [77,114], can be found together with genotype-specific compounds, such as kalanchosides [115] and kalanhybrins [71].

As cardiac glycosides, the original bioactivity attributed to bufadienolides is their cardiotonic activity, acting as inhibitors of the sodium pump at the myocardial tissue [116]. However, its reduced therapeutic window conditions its efficacy, allowing eventual cardiotoxic events due to overdosage [117]. In fact, the accidental consumption of *Bryophyllum* species by different mammals is one of the leading causes of cattle mortality in Africa [118], with reporting episodes of stroke, subendocardial hemorrhages, and heart tissue necrosis [119]. The biosynthesis of bufadienolides is a plant defensive mechanism against insect and herbivore attacks. They have already been reported as effective insecticidal compounds [31].

Bufadienolides have also been described as potent anticancer agents, as demonstrated by a number of in vitro studies with multiple cancer cell lines (Table 4) [120]. Nevertheless, their inherent toxicity difficult their administration in animal and human models [121]. Current research on these compounds is focused on finding effective and safer semi-synthetic derivatives [122].

Table 4 shows the associated bioactivities of identified bufadienolides in *Bryophyllum* sp., with a special focus on the cytotoxic activity of these compounds, being effective against relevant cancer cell lines, mainly those derived from breast, ovarian and lung carcinomas [71,115].

The bioactivity of phenolic and bufadienolides compounds reveals an unexploited phytochemical potential associated with *Bryophyllum* sp. However, research on these secondary metabolites is still very limited, since their concentration and activity depend on adaptive responses of plants, which is why low-yield extraction protocols have been reported [61,123]. Consequently, in order to explore the phytochemical properties of these medicinal plants, the establishment of efficient biotechnological approaches is required to achieve the valorization of *Bryophyllum* subgenus.

## 3. Plant Tissue Culture for Sustainable Valorization of Bioactive Compounds of *Bryophyllum* sp.

Currently, medicinal plants represent the source of more than 25% of drugs officially approved by the Food and Drug Administration (FDA) and the European Medicinal Agency (EMA) for the development of novel synthetic drugs [128]. Their derived by-products account for the 75–90% of the total used in the primary healthcare systems of economically developed nations [129]. However, only 6% of the plants have been studied from the pharmacological point of view and for 85% of them their phytochemical potential has not been evaluated [130], which represents a vast territory of families of plants with medicinal properties unexplored, such as *Bryophyllum* sp. Novel strategies, based on plant biotechnology methodologies, are required to meet the growing global demand for products derived from medicinal plants for industrial purposes in different sectors, such as the food, cosmetic, and pharmaceutical industries [131].

**Figure 3:**
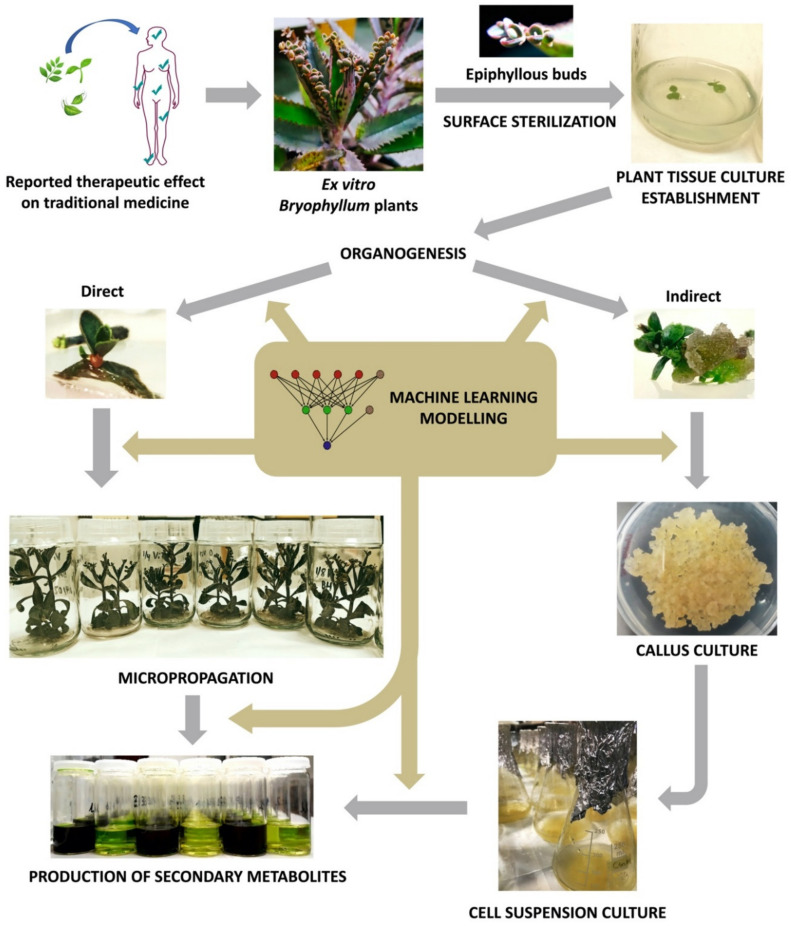
Workflow diagram of *Bryophyllum* sp. valorization via plant tissue culture (PTC).

Since then, plant biotechnology has constantly evolved, and it currently provides a reliable methodology for the bioproduction of secondary metabolites with pharmacological value, by using plant in vitro systems [132]. Consequently, plant tissue culture (PTC) became a basic biotechnological methodology with countless applications in different areas of knowledge [133]. However, PTC must face its own limitations, as it involves a set of highly specialized, usually expensive, techniques that are extremely sensitive to multiple factors [134]. In this section, we will provide a deeper insight about the key aspects of PTC, with particular focus on the methodology applied to *Bryophyllum* sp. (Figure 3).

### 3.1. PTC Establishment

The first step of a PTC protocol is the effective removal of pathogenic contaminants from the selected plant material (Figure 3) [135]. Therefore, the sterilization of the explant surface is required through a procedure that ensures convenient disinfection while maintaining its integrity, along with aseptic handling in laminar flow cabinets [136].

In particular, our research group has developed a simple and reliable method for the disinfection of epiphyllous buds of the adult plants of *B. daigremontianum*, *B. × houghtonii* and *B. tubiflorum* species grown in greenhouse, which involves the use of common, safe and environmental-friendly disinfectant agents [62,64,80,101,137]. The protocol includes an initial tap water wash of the buds overnight, followed by a two-step stage, where the buds are rinsed in 70% ethanol (*v*/*v*) for 1 min, washed with sterile distilled water and then rinsed in 0.4% (*v*/*v*) sodium hypochlorite with a few drops of Tween^®^-20 for 10 min. Finally, buds are gently washed with sterile distilled water and dried to remove persistent residues of disinfection agents. After the establishment under aseptic conditions, disinfected buds are placed and cultured in growth chambers under controlled conditions of photoperiod and temperature, thus enabling an adequate culture development. This procedure represents an improvement on the previously established disinfection protocols for *Bryophyllum* sp., in which slower procedures were performed [138] with more concentrated disinfectants [139]; with greater losses of viability [140] and explants integrity [141], or with more polluting agents, such as mercury chloride [142].

#### Plant Culture Media Composition

Plant culture media composition plays a crucial role in the success of PTC protocols, as the nutrition of cultured plant materials depends directly on its ingredients [143]. As a general rule, plant culture medium formulations contain a series of inorganic nutrients, divided into macro- and micronutrients according to their requirements for plant physiology, along with organic nutrients, such as vitamins [144]. Among the countless culture media formulations defined in PTC protocols, the formulation described by Murashige and Skoog in 1962 [145], mostly known as MS medium, is considered the universal medium to be applied as standard for different plant biotechnological applications [146]. The universality of the MS medium is based on its high levels of nitrogen sources, with a relatively high ratio of ammonium to nitrate [147]. However, it has recently been pointed out that the composition of the MS medium is supra-optimal for some species and therefore harmful due to an excessive concentration of ammonium ions [148,149].

*Bryophyllum* sp. are especially affected by excess of ammonium ion. This cation negatively affects the growth of these species, due to a deterioration of CAM photosynthetic efficiency [150,151]. Although PTC of *Bryophyllum* sp. has been established using MS medium [138,139], better growth and multiplication rates were achieved when the composition of the MS medium was modified, as it has been shown by reducing the concentration of macronutrients by half for *B. daigremontianum*, *B. × houghtonii* and *B. tubiflorum* [64,80,101].

### 3.2. Organogenesis and Plant Regeneration

Thus far, the information on the plant regeneration protocols for *Bryophyllum* sp. is limited. Most publications focus on the establishment of indirect regeneration protocols [138,139,140,141,152]. Recently, we have provided information on the effect of exogenous application of plant growth regulators (PGRs) on the in vitro organogenesis of *B. daigremontianum*, *B. × houghtonii* and *B. tubiflorum* [137], pointing at the concentration of the cytokinin 6-benzylaminopurine (BAP) as the most critical factor guiding this process. Specifically, it was demonstrated that at operational concentrations of BAP (0.375–0.75 mg L^−1^) both *B. daigremontianum* and *B. × houghtonii* present a higher frequency of direct shoot regeneration than *B. tubiflorum*. In turn, *B. tubiflorum* was revealed as the most efficient species for the induction of callus formation during indirect organogenesis [137]. These results highlight the complexity of the design of plant in vitro regeneration protocols and shed light into the organogenesis-related processes of *Bryophyllum* sp., facing to the large-scale exploitation of these medicinal plants.

### 3.3. Micropropagation

After the establishment of axenic cultures, PTC protocols normally are followed by multiplication, rooting and acclimatization stages, throughout the procedure called micropropagation (Figure 3), with the objective of achieving a large number of fully-developed true-to-type individuals [135]. The singular asexual reproduction that takes place at leaf margins of *Bryophyllum*, results in the clonal propagation of fully-developed epiphyllous buds, presenting individual aerial and root systems [11,12]. For this reason, *Bryophyllum* constitutes an outstanding subgenus for the micropropagation of different species. Nevertheless, the micropropagation of *Bryophyllum* is not exempt from difficulties due to its particular metabolism and poor nutritional requirements [151]. In this sense, it was recently reported that ammonium, sulfur, molybdenum, copper, and sodium play a crucial role on growth and plantlet formation on in vitro-cultured *Bryophyllum* in a species-dependent manner [153]. Therefore, multiple nutritional modifications may be required to achieve genotype-specific optimization, since mineral imbalances and interactions could directly influence the success of PTC protocols, by affecting micropropagation performance [154], and causing undesirable physiological disorders [148].

### 3.4. Establishment of Plant Suspension-Cultured Cells (PSCCs)

In the last decades, an increasing interest of plant biotechnology has been addressed to the evaluation and valorization of medicinal plants, with the aim of exploring their phytochemical potential and making it accessible to industrial applications [155]. In order to maximize the advantages of PTC for the production of secondary metabolites, plant suspension-cultured cells (PSCCs) emerged as a valuable biotechnological platform [156].

A single recent report is available for the establishment of PSCCs from *B. × houghtonii* [101]. In this work, the use of PSCCs from *B. × houghtonii* for the production of bioactive compounds was reported, with a special focus on the operational aspects required for the establishment of plant cell cultures, such as the determination of growth kinetics [101]. The typical four-phase growth behavior was reported only after 8 days of culture, starting with an initial lag phase where cells acclimatize to new culture conditions and no growth is observed. The lag phase is followed by the exponential phase, where cell divisions occur massively, reported by a severe increase in cell biomass. Afterward, a stationary phase is reached: cell growth stabilizes and the accumulation of secondary metabolites is observed, before reaching death phase, in which cell death takes place due to a lack of nutrients [157].

PSCCs are considered a valuable biological platform for the application of several approaches to enhance plant secondary metabolism, which have been widely exploited in the field of plant biotechnology for the production of bioactive compounds: elicitation, precursor feeding, two-phase culture system, and metabolic engineering [158]. Among them, elicitation is the most extended approach applied to PSCCs, although it can be applied to many other culture types [159]. Due to the importance of elicitation on the evaluation of medicinal plants and their phytochemical potential, the next section will be focused on this phenomenon, with a particular focus on the elicitation of *Bryophyllum* sp.

### 3.5. Enhancement of Phenolic Compounds Production from Bryophyllum sp. via Elicitation

In the last years, great efforts regarding the improvement of plant secondary metabolism have been made in the field of plant biotechnology, being the elicitation of PSCCs one of the most successful approaches applied for the large-scale production of plant bioactive compounds [160]. A review of the literature shows that the number of publications selected by Google Scholar^®^ from the search “elicitation of plant cell culture” is close to 15,000 entries in the last five years.

The term elicitation, as recently defined by Narayani and Srivastava (2017) [161], refers to “the manipulation of biochemical and metabolic pathways, via stress induction, that can be implemented for enhancing secondary metabolite production and characterize the role of stress factors on plants using plant cell and/or tissue cultures as model systems”. On this basis, different types of culture may constitute precious biological platforms for the stimulation of plant secondary metabolism under controlled conditions, by the administration of elicitors (Figure 3). In all cases, obtaining the maximum viability and integrity of the elicited cultures is required in order to achieve an efficient and sustainable production system [162].

Little information about the elicited production of bioactive compounds by *Bryophyllum* sp. can be found in the literature. Recently, the elicitation of phenolic compounds from in vitro-cultured *Bryophyllum* sp. subjected to nutritional stress has been reported by García-Pérez and co-workers (2020) [62]. It was found that a decrease in the ammonium concentration in the culture medium causes a 50% overproduction and accumulation of phenolic compounds in the aerial parts of *B. × houghtonii*. The effect was less in magnitude in *B. daigremontianum* and *B. tubiflorum* [62]. In addition, the antioxidant efficiency of the derived *Bryophyllum* extracts was assessed in terms of their free-radical scavenging activity and lipid peroxidation inhibition [62,64], suggesting that in vitro-cultured *B. × houghtonii* can be considered a medicinal species with an improved phytochemical potential [80], in comparison to closely-related species, such as *B. daigremontianum* and *B. tubiflorum*. In this sense, due to its phytochemical potential, PSCCs from *B. × houghtonii* were subjected to elicitation by cyclodextrins (CDs) [101]. CDs are cyclic oligosaccharides able of forming inclusion complexes with hydrophobic molecules. The results suggested that CDs elicited the production of phenolic compounds in *Bryophyllum* PSCCs, as well as their associated free-radical scavenging activity. Specifically, it was shown that CDs favored the accumulation of total phenols and flavonoids in the culture medium (7.9 and 17.3-fold increases, respectively) after 7 days of culture, thus, preserving the integrity of the cellular fraction for subsequent elicitation cycles [101].

Altogether, the application of novel approaches should be developed in order to reveal the full phytochemical potential of *Bryophyllum* sp., based on the application of unexploited PTC strategies, taking benefit of the countless advantages provided by PSCCs, committed to the enhancement of plant secondary metabolite production.

## 4. Machine Learning for Optimizing the Biotechnological Valorization of *Bryophyllum* sp.

Along with this review, we provided evidence about the multifactorial behavior of PTC methodologies and the production of secondary metabolites (Figure 3). Therefore, the elucidation and characterization of such phenomena may require the development of complex experimental designs, to reveal relevant interactions between factors, which are not feasible due to cost and time. Furthermore, the analysis and interpretation of these complex experimental designs is difficult and, in many cases, limited or incomplete [163].

Machine Learning (ML) techniques stand out as a cutting-edge alternative to detect the critical factors behind a certain procedure, as well as a method to establish the influence of possible interactions between them [164]. The application of ML algorithms allows the modeling of complex processes, a powerful tool for making decisions and studying unknown phenomena [165]. Among the different ML tools, the combination of artificial neural networks with fuzzy logic, commonly known as neuro-fuzzy logic (NFL), constitutes a robust computational tool for the optimization and prediction of complex processes [166]. Furthermore, NFL offers another advantage regarding the efficacy of predictive models, thus providing direct knowledge from a detailed interpretation of the results, by the establishment of simple “IF-THEN” rules, that facilitate making conclusions [167].

Concerning *Bryophyllum* sp., the application of NFL was already applied to the identification of critical factors involved in plant in vitro nutrition [153] and organogenesis [137], as well as the production of phenolic compounds [62].

In this sense, ML was able to identify the key mineral nutrients and their interactions, in order to optimize the growth and reproduction of *Bryophyllum* sp. cultured in vitro. Among the 18 different mineral nutrients used on MS formulation, ML detected that only five nutrients were critical on *Bryophyllum* in vitro culture, in a genotype-dependent manner [153]. Specifically, ammonium, sulfate, sodium, molybdenum, and copper were selected by NFL as the critical factors guiding several growth-related parameters, and the interaction between sulfate and molybdenum was widely reported as responsible for most parameters: root length, plantlet formation, and aerial parts fresh weight [153].

ML was also employed for the modeling and predicting of *Bryophyllum* organogenesis in vitro [137]. BAP concentration was assessed as the critical factor guiding this phenomenon on B. daigremontianum, B. × houghtonii and B. tubiflorum; thus, predicting a minimal BAP concentration required for the development of different organogenetic responses (0.35 mg L^−1^). On the contrary, the application of auxins, such as indoleacetic acid (IAA), was outlined as an inhibitory factor on the indirect shoot regeneration on B. tubiflorum, whereas no IAA influence was reported on *B. daigremontianum* and *B. × houghtonii* [137].

Additionally, the production of phenolic compounds by in vitro-cultured *Bryophyllum* sp. was optimized using ML [62]. It was observed that phenolic compounds accumulation achieved the maximum concentrations in the aerial parts of cultured plants under low ammonium concentrations (<15 mM). Moreover, the extraction of total phenolic compounds was enhanced by the use of 55–85% aqueous methanol, whereas flavonoids were mostly extracted with higher methanol concentrations in water (>85%). In addition, the antioxidant potential of *Bryophyllum* extracts, in terms of radical-scavenging activity, was shown to be improved using 55–85% MeOH as solvent on *B. × houghtonii* cultured under low ammonium concentrations [62]. Furthermore, these experimental conditions for maximizing the antioxidant activity of *B. × houghtonii* were also validated in terms of preventing lipid oxidation [64] and plant in vitro growth [153]; thus, assessing the effectiveness of ML on the valorization of *Bryophyllum*.

## 5. Concluding Remarks and Future Perspectives

This review focuses on the ethnomedicinal uses of *Bryophyllum* sp., and their main phytoconstituents, responsible for its biological and pharmacological properties (cytotoxic, sedative, insecticidal, antiviral, and anticancer activities).

It has also been described how the development of optimal methods of plant tissue culture for *Bryophyllum* sp. can lead to the valorization of the bioactive compounds of these species, which are highly promising in therapeutics as antioxidant and anti-tumor agents. In this sense, the application of machine learning technology to model and optimize the production of phytochemicals is of extraordinary interest as it could favor their large-scale biotechnological exploitation in the cosmetic, food, and pharmaceutical industries.

In our opinion, the current trends and future challenges on the research of non-well characterized medicinal plants are: (i) the combination of plant in vitro systems, using molecular approaches, and nutritional studies [128], with the purpose of characterizing their full biosynthetic potential; (ii) the enhancement of secondary metabolism throughout the application of metabolic engineering, as it has been performed in the cases of *trans*-resveratrol [168] and terpenoid indole alkaloids [169]; (iii) the application of high throughput technologies, such as metabolomics, for the characterization of metabolic fingerprinting associated to elicitation of PSCCs, in response to plant stress, at a biochemical and biosynthetic level [170,171]. Finally, the combination of PTC and ML with omics technologies will assist to the establishment of new perspectives on the field of phytochemistry and the study of the biosynthetic potential of unexplored plants, as it is the case of *Bryophyllum*. Nowadays, the cornerstone in clinical management includes two easy concepts that are dietary modification associated with improvement of physical activity, also defined as lifestyle change [172,173] through a revaluation to popular medicine.

## Figures and Tables

**Table 1:** Ethnobotanical uses of Bryophyllum species.

Species	Ethnobotanical Uses	Plant Organ	Locations ^1^	References
*B. crenatum* (Andr.) Baker	Wounds, smallpox, otitis, cough, asthma, palpitations, headache, abscesses, convulsions, general debility, diabetes, obstetrics and gynecology, vermifuge, abortion, antimicrobial treatment	Leaves Roots	Africa	[32,33,34,35]
*B. daigremontianum* Raym.-Hamet et Perr.	Leucorrhea, dysmenorrheal, carminative, psychic agitation, anxiety, restlessness	Leaves	Bangladesh	[28,36]
*B. fedtschenkoi* Raym.-Hamet et Perr.	Analgesic, cytotoxic, antimicrobial treatment	Leaves Aerial parts Woody stems	Brazil	[37,38,39]
*B. mortagei* (Raym.-Hamet et Perr.) G.E. Wickens	Digestive disorders, neoplastic diseases, vermifuge, antimicrobial treatment	Aerial parts Flowers Roots	Mexico, Colombia, Indonesia	[37,40,41,42]
*B. pinnatum* (Lam.) Oken	Wounds, burns, coughs, earache, headache, muscle pain, asthma, bronchitis, pneumonia, arthritis, rheumatism, ulcers, diabetes, urinary bladder stones, dysentery, diarrhea, vermifuge, antibacterial, insect bites, fevers, menstrual disorders, nausea, tumors, gynecology	Leaves Roots	Nigeria, Uganda, Madagascar, India, China, Vietnam, Bangladesh, Australia, Brazil, Peru, Trinidad and Tobago	[43,44,45,46,47,48,49,50,51,52]
*B. serratum* (Mann. and Boit.) Blanco	Pain, inflammation, fever, antiviral	Stems	Taiwan	[53,54]
*B. tubiflorum* Harv.	Wounds, epilepsy, vermifuge, neoplastic diseases	Leaves	Brazil, Ethiopia	[29,30]

^1^ Locations where the ethnobotanical uses have been reported.

**Table 2:** Phenolic acids reported in *Bryophyllum* sp.

Subfamily	Compound ^1^	Species ^2^	References
Cinnamic acids	p-Coumaric acid	BD, BP, BT	[63,82,88,89,90]
	Caffeic acid	BD, BP, BT	[63,79,88,91]
	Chlorogenic acid	BD, BT	[63,92]
	Ferulic acid	BD, BP, BT	[26,63,82,92,93]
Benzoic acids	p-Hydroxybenzoic acid	BD, BP, BT	[91]
	Protocatechuic acid	BD, BP, BT	[26,63,82,91,93]
	Vanillic acid	BT	[58,78]
	Gallic acid	BD, BP, BT	[63,78,82,88,90,91,93]
	Syringic acid	BD, BP, BT	[63,78,90]

^1^ Compounds are named as their free-form to simplify the identification. ^2^ BD: *B. daigremontianum*; BP: *B. pinnatum*; BT: *B. tubiflorum*.

**Table 3:** Flavonoids reported in *Bryophyllum* sp.

Subfamily	Compound ^1^	Species ^2^	References
Flavanones	Naringenin	BT	[92]
Flavones	Luteolin	BP	[89,94,111]
	Apigenin	BP, BT	[50,78]
	4’,5-dihydroxy-3’,8-dimethoxyflavone	BP	[109,112]
	Acacetin	BP	[90]
	Diosmetin	BP	[90]
	Afzelin	BP	[102]
	Galangustin	BT	[58]
	Hispidulin	BT	[92]
Flavonols	Quercetin	BD, BP, BT	[58,77,78,88,89,92,94,95,109]
	Kaempferol	BD, BP, BT	[77,78,88,89,90,92,102,109,112]
	Quercitrin	BP	[109,112]
	Myricetin	BD, BP, BT	[77,90,92]
	Rutin	BP	[89,94]
	Isorhamnetin	BD, BP	[77,88]
	Kaempferitrin	BP	[102]
	Herbacetin	BT	[58]
	Patuletin	BD	[77]
	Isoquercetin	BT	[92]
	Aromadendrin	BT	[92]
	Galangin	BT	[92]
Flavanols	Catechin	BP	[89]
	Epicatechin	BT	[92]
	Epigallocatechin	BP	[111]

^1^ Flavonoids are named as their free-form to simplify the identification. ^2^ BD: *B. daigremontianum*; BP: *B. pinnatum*; BT: *B. tubiflorum*.

**Table 4:** Bufadienolides identified in *Bryophyllum* sp. and their associated bioactivities.

Species ^1^	Plant Organ	Bufadienolides	Bioactivities ^2^	References
BD	Roots	11α,19-dihydroksytelocinobufagin, bersaldegenin-1-acetate, bersaldegenin-1,3,5-orthoacetate, 19-(acetyloxy)-3β,5β,11α,14-tetrahydroxyl-12-oxo-bufa-20,22-dienolide and 19-(acetyloxy)-1b,3b,5b,14-tetrahydroxyl-bufa-20,22-dienolide	Moderate antioxidant activity using in vitro blood plasma model under peroxynitrite-induced oxidative stress. Effective for prevention of lipid hydroperoxides generation and thiobarbituric acid-reactive substances (TBARS)	[24]
BP	Leaves	Bryophyllin A and C	Insecticidal against silkworm larvae	[66]
BH	Leaves	Bryophyllin A and C, bersaldegenin-1-acetate, bersaldegenin-3-acetate, bersaldegenin-1,3,5-orthoacetate, daigremontianin, methyl daigremoniate	Insecticidal against silkworm larvae, except for bersaldegenin-1-acetate. Cytotoxic effect of bersaldegenin-1,3,5-orthoacetate and daigremontianin against induced Raji cell line (Burkitt’s lymphoma); inhibition of Epstein–Barr virus	[31,67]
BH	Whole plant	Kalanhybrins A, B and C, bersaldegenin-1-acetate, bersaldegenin-3-acetate	Cytotoxic activity of bersaldegenin derivatives against human breast MCF-7 cancer cell line, human lung carcinoma NCI-H460 and glioblastoma SF-268 cell line	[71]
BD	Roots	Kalandaigremosides A-H	nd	[124]
BP	Whole plant	Bryophyllin A and B, bersaldegenin-3-acetate	Cytotoxic effect against keratin-forming tumor KB cell line, adenocarcinomic human alveolar basal epithelial A-549 cell line and human ileocecal carcinoma HCT-8 cell line	[125]
BP, BD, BT	Leaves (BD, BP) and stems (BT)	BP, BT: bersaldegenin-1-acetate, bersaldegenin-3-acetate, bersaldegenin-1,3,5-orthoacetate, bryophyllin A. BD: Bersaldegenin-1,3,5-orthoacetate	nd	[114]
BD	Leaves	Bersaldegenin-1,3,5-orthoacetate, daigremontianin	Insecticidal against silkworm larvae	[65]
BP	Leaves	Bersaldegenin-1-acetate, bersaldegenin-3-acetate, bersaldegenin-1,3,5-orthoacetate, bryophyllin A	nd	[90]
BD, BP	Leaves	BD: Bersaldegenin-1-acetate, bersaldegenin-2-acetate, bersaldegenin-1,3,5-orthoacetate, bryophyllin A, daigremontianin. BP: Bersaldegenin-1-acetate, bersaldegenin-2-acetate, bersaldegenin-3-acetate, bersaldegenin-4-acetate, bersaldegenin-5-acetate, bersaldegenin-1,3,5-orthoacetate, bryophyllin A	Cytotoxic activity against human ovarian cancer SKOV-3 cell line, cervical adenocarcinoma HeLa S3 cell line and malignant melanoma A375 cell line. Antimicrobial activity against *Corynebacterium diphtheriae*, *Staphylococcus aureus*, *Staphylococcus epidermidis,* and *Enterococcus hirae*	[77,126]
BT	Whole plant	Kalantubosides A and B, bryophyllin A, bersaldegenin-1-acetate, bersaldegenin-1,3,5-orthoacetate	Cytotoxic effect against adenocarcinomic human alveolar basal epithelial A-549 cell line, promyelocytic leukemia HL-60 cell line, oral adenosquamous carcinoma Cal-27 cell line, and melanoma A2058 cell line	[127]

^1^ BD: *B. daigremontianum*; BH: *B. × houghtonii*; BP: *B. pinnatum*; and BT: *B. tubiflorum*. ^2^ nd: not determined.
